# Immunologic risk stratification of pediatric heart transplant patients by combining HLA-EMMA and PIRCHE-II

**DOI:** 10.3389/fimmu.2023.1110292

**Published:** 2023-03-14

**Authors:** M. Ellison, M. Mangiola, M. Marrari, C. Bentlejewski, J. Sadowski, D. Zern, Cynthia Silvia Maria Kramer, S. Heidt, M. Niemann, Q. Xu, A. I. Dipchand, W. T. Mahle, J. W. Rossano, C. E. Canter, T. P. Singh, W. A. Zuckerman, D. T. Hsu, B. Feingold, S. A. Webber, A. Zeevi

**Affiliations:** ^1^ University of Pittsburgh Medical Center, Histocompatibility Laboratory, Pittsburgh, PA, United States; ^2^ Transplant Institute, NYU Langone Health, New York University, New York, NY, United States; ^3^ Department of Pathology, School of Medicine, University of Pittsburgh, Pittsburgh, PA, United States; ^4^ Department of Immunology, Leiden University Medical Center (LUMC), Leiden, Netherlands; ^5^ Research and Development, PIRCHE AG, Berlin, Germany; ^6^ Labatt Heart Centre, Hospital for Sick Children, University of Toronto, Toronto, ON, Canada; ^7^ Children’s Healthcare of Atlanta, Emory University, Atlanta, GA, United States; ^8^ Division of Cardiology, Children’s Hospital of Philadelphia, Philadelphia, PA, United States; ^9^ Division of Cardiology, Department of Pediatrics, School of Medicine, University of Washington, Seattle, WA, United States; ^10^ Department of Cardiology, Boston Children’s Hospital, Harvard Medical School, Boston, MA, United States; ^11^ Columbia University, Irving Medical Center, New York, NY, United States; ^12^ Division of Pediatric Cardiology, Children’s Hospital at Montefiore, Albert Einstein College of Medicine, Bronx, New York, NY, United States; ^13^ Department of Pediatrics, Children’s Hospital of Pittsburgh, Pittsburgh, PA, United States; ^14^ Department of Pediatrics, Vanderbilt University Medical Center, Nashville, TN, United States

**Keywords:** PIRCHE-II, HLA-EMMA, donor specific antibody, antibody mediated rejection (ABMR), pediatric heart transplantation

## Abstract

Human leukocyte antigen (HLA) molecular mismatch is a powerful biomarker of rejection. Few studies have explored its use in assessing rejection risk in heart transplant recipients. We tested the hypothesis that a combination of HLA Epitope Mismatch Algorithm (HLA-EMMA) and Predicted Indirectly Recognizable HLA Epitopes (PIRCHE-II) algorithms can improve risk stratification of pediatric heart transplant recipients. Class I and II HLA genotyping were performed by next-generation sequencing on 274 recipient/donor pairs enrolled in the Clinical Trials in Organ Transplantation in Children (CTOTC). Using high-resolution genotypes, we performed HLA molecular mismatch analysis with HLA-EMMA and PIRCHE-II, and correlated these findings with clinical outcomes. Patients without pre-formed donor specific antibody (DSA) (n=100) were used for correlations with post-transplant DSA and antibody mediated rejection (ABMR). Risk cut-offs were determined for DSA and ABMR using both algorithms. HLA-EMMA cut-offs alone predict the risk of DSA and ABMR; however, if used in combination with PIRCHE-II, the population could be further stratified into low-, intermediate-, and high-risk groups. The combination of HLA-EMMA and PIRCHE-II enables more granular immunological risk stratification. Intermediate-risk cases, like low-risk cases, are at a lower risk of DSA and ABMR. This new way of risk evaluation may facilitate individualized immunosuppression and surveillance.

## Introduction

Disparity in human leukocyte antigen (HLA) matching has been used to assess heart transplant risk, and it has been shown that patient outcomes improve with an increasing degree of antigen match (1). Molecular level HLA compatibility has been gaining popularity as a method for obtaining a more detailed assessment of HLA mismatch between donor and recipient (2, 3). Currently, various algorithms exist for molecular level HLA mismatch assessment; HLAMatchmaker (4), HLA Epitopes Mismatch Algorithm (HLA-EMMA) (5), Predicted Indirectly Recognizable HLA Epitopes (PIRCHE-II) (6), and the Electrostatic Mismatch Score (EMS) (7, 8). HLAMatchmaker considers patches of polymorphic amino acid residues, termed eplets, to compare donor and recipient to predict B-cell humoral response (4). HLA-EMMA attempts to predict humoral response by determining the number of amino acid mismatches (AAMM) and solvent-accessible amino acid mismatches (SAAAMM) (5). In contrast, PIRCHE-II takes a different approach to predict T-cell response by determining the number of donor HLA peptides indirectly presented by the recipients’ Class II HLA to recipient CD4 T-cells (6). Finally, the EMS algorithm scores HLA amino acid mismatches based on their physiochemical properties. Here we focus our attention on HLA-EMMA and PIRCHE-II (7, 8).

Mechanisms of allograft rejection include both direct and indirect allorecognition (9–11). The T- and B-cell response to non-self occurs in a cooperative manner during the process of allorecognition (12–16).This orchestrated cellular cooperation leads us to our working hypothesis that a combination of algorithms that predict both T-cell and B-cell response to an allograft will better predict risk of donor specific antibody (DSA) formation and antibody mediated rejection (ABMR) than the use of a single algorithm that predicts either T-cell or B-cell response.

Our previous work illustrated how HLAMatchmaker and PIRCHE-II can be used together to identify patients at low risk of developing DSA and ABMR despite a high-risk score with the individual algorithms (17). To further validate our hypothesis that using a combination of T and B cell response prediction algorithms offers a better means to risk stratify patients, we performed additional analyses using a different B-cell response prediction algorithm: HLA-EMMA.

We expect HLA-EMMA to achieve similar results to HLAMatchmaker, but it is important to prove that this is true and that HLA-EMMA can be combined with PIRCHE-II to good effect because many laboratories use the algorithm in place of HLAMatchmaker. It is also important to define cutoffs for HLA-EMMA and determine how the algorithm performs on its own for this cohort. At the writing of this article 178 software licenses for HLA-EMMA have been provided to labs in at least 22 countries across the world. There are currently no pediatric heart transplant studies and only one adult heart transplant study that makes use of the HLA-EMMA algorithm (18). HLA-EMMA is growing in popularity; thus, it is important to establish score cutoffs for risk stratification using accurately typed cohorts.

Here we demonstrate how HLA-EMMA performs both alone and in combination with PIRCHE-II in a pediatric heart transplant cohort on which we used the most accurate form of HLA typing available. Our investigation demonstrates that HLA-EMMA can predict risk of DSA and ABMR and establishes risk cutoffs for HLA Class I, DR and DQ mismatches. We also show that HLA-EMMA and PIRCHE-II can be used together to risk-stratify patients and identify patients at high, intermediate, and low risk of DSA and ABMR.

## Materials and methods

### Study cohort

The study cohort consisted of 274 pediatric heart transplant recipients enrolled in the Clinical Trials in Organ Transplantation in Children (CTOTC) with available recipient and donor DNA. All patients in the CTOTC trial were chronologically recruited by physicians at participating centers over a period of two years and were treated using the same immunosuppressant protocol (19). This cohort provided HLA genotyping data to generate descriptive statistics for HLA-EMMA and PIRCHE-II. One-hundred thirty-one cases had clinical data for DSA and ABMR. After excluding 31 cases with pre-formed DSA, the remaining 100 cases were used to determine cut-off values (molecular mismatch thresholds) for the risk of DSA and ABMR. Characteristics of this 100-patient cohort are described in **Table 1** and are similar to previously reported characteristics of a large portion of the CTOTC study’s patients (20). The 100 patients have a mean follow up time of 3 years post-transplant. Descriptive statistics for HLA-EMMA and PIRCHE-II were generated for the entire cohort of 274 patients with genotype. The 143 cases for whom we do not currently have access to the clinical data will be used to validate the cut-off values generated in this pilot study. A flow diagram of this study cohort was previously published (see **Figure 1** of Mangiola et al., 2022) (17).

**Table 1:** Baseline characteristics for the 100 patients used in HLA molecular mismatch analysis and correlation with DSA and ABMR outcomes.

Characteristic	Total (n=100)
Age	
Median age at listing, years (IQR)	5.49 (0.57, 13.76)
Median age at transplant, years (IQR)	5.66 (0.68, 13.59)
Weight	
Median weight at listing (IQR)	18.5 (6.9, 46)
Median weight at transplant (IQR)	15.5 (6, 46.2)
Diagnosis	
Cardiomyopathy	60
Congenital heart disease	28
Cardiomyopathy and Congenital heart disease	4
Other	1
Race	
White	53
Black	26
Asian	7
Unknown or not reported	7
Sex	
Male	46
Female	47
Blood type	
A	26
AB	2
B	17
O	48
UNOS status at listing	
1A	62
1B	17
2	14
UNOS status at transplant	
1A	83
1B	7
2	3
Prior sensitizing event	
Surgery	41
Blood transfusion	41
VAD	15
ECMO	6
Any MCS	21
Homograft	9
Prior transplant	2
Pregnancy	1
Hospitalized at listing	70
ICU at listing	42
Ventilator at listing	12
ECMO at listing	1
VAD at listing	10
MCS at listing	19
Hospitalized at transplant	69
ICU at transplant	39
Ventilator at transplant	10
ECMO at transplant	4
VAD at transplant	17
MCS at transplant	21

ECMO, extracorporeal membrane oxygenation; ICU, intensive care unit; IQR, interquartile range; MCS, mechanical circulatory support; UNOS, United Network for Organ Sharing; VAD, ventricular assist device. Note that 7 of the 100 patients lacked demographic information. All values reported as n unless otherwise noted. HLA molecular mismatch data for this cohort is summarized in **Table 2** and Mangiola et al., 2022.

**Figure 1:**
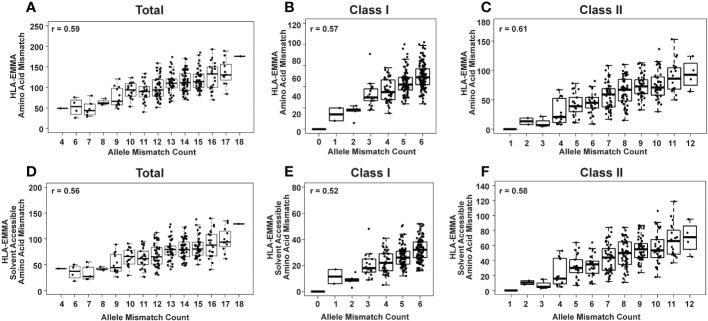
HLA-EMMA total amino acid mismatch (AAMM) **(A-C)** and solvent-accessible amino acid mismatch (SAAAMM) **(D-F)** for 274 pediatric heart transplant cases. Graphs show mismatches for all HLA loci tested: Class I + Class II **(A, D)** Class I **(B, E)**, and Class II **(C, F)**. The Pearson’s correlation coefficient rho (r) is stated on each plot.

### Ethics

Approval from the IRB for the CTOTC studies was obtained for all clinical sites and informed consent was obtained based on each participating center’s IRB-approved consent form. When appropriate based on age and developmental maturity, assent was obtained. The study is registered at ClinicalTrials.gov (NCT02752789). No animal research was performed.

### HLA genotyping

Class I and Class II HLA typing (HLA-A, B, C, DRB1/3/4/5, DQA1, DQB1, DPA1, DPB1) was performed by next generation sequencing (NGS) using Immucor MIA FORA NGS Flex 11 Kit (Immucor^®^, Peachtree Corners, GA) according to the manufacturer’s instructions. Sequencing was conducted on an Illumina MiSeq^®^ using a standard v2 flow cell and a 300-cycle MiSeq^®^ V2 reagent kit (Illumina, San Diego, CA). Sequencing data in FASTQ format were demultiplexed and analyzed using MIA FORA HLA Flex software v4.5 with IMGT database 3.36.0 (Immucor). Ambiguities were resolved by sequence-specific oligonucleotide and/or sequence-specific primer genotyping.

### HLA molecular mismatch determination

HLA allele and antigen matching was conducted by comparing donor and recipient typing results after null alleles had been excluded from the dataset. AAMM and SAAAMM scores were determined for each HLA locus using HLA-EMMA version 1.04 (https://hla-emma.com), which predicts B-cell response. HLA-EMMA AAMM score is determined by matching HLA proteins using their individual amino acid residues with mismatches in the donor relative to the recipient being counted to produce a score. HLA-EMMA SAAAMM score is the number of amino acids included in the AAMM score that are solvent accessible (5). Locus scores were summed to calculate Class I (HLA-A+HLA-B+HLA-C), DR (HLA-DRB1+HLA-DRB3+HLA-DRB4+HLA-DRB5) and DQ (HLA-DQA1+HLA-DQB1) AAMM and SAAAMM scores.

PIRCHE-II scores were determined for each HLA locus using the PIRCHE-II algorithm (https://www.pirche.com, version 3.3.57 with database version 3.40) and summed (as stated for HLA-EMMA above) to achieve Class I, DR and DQ scores. PIRCHE-II is a prediction algorithm that estimates the amount of non-self, donor-derived HLA peptides that can be presented by recipient HLA-DRB1/3/4/5, HLA-DQA1/HLA-DQB1, and HLA-DPA1/HLA-DPB1 antigens to the recipient’ CD4 T cells. The number of peptides from the donor containing amino acid mismatches to the recipient that can be presented by recipient determine the PIRCHE-II score (6).

Quantile analysis was used to determine cut-offs for the various algorithms. This process uses the distribution of scores from HLA-EMMA or PIRCHE-II for DSA or ABMR cases only, and determines a position in the distribution (cut-off) at which 95% of patients with the outcome of interest (DSA or ABMR) have a score above this position and 5% score below. This is done by identifying the 0.05 position in the probability distribution. The numerical value of this position is used as the cut-off for risk of DSA or ABMR for a given HLA antigen.

### HLA antibody analysis

The CTOTC core antibody laboratory (University of Pittsburgh) performed all patient serum testing using the Luminex LABScreen^®^ Single Antigen (One Lambda, Thermo Fisher, West Hills, CA) system, as previously described (19, 21). Testing was performed at ten timepoints: at enrollment, while on waitlist, immediately pre-transplant, 7 days, 1, 3, 6, 12, 24, and 36 months post-transplant. Additional testing was performed at the time of predefined clinical events such as acute rejection. A positive result for post-transplant DSA was defined as ≥1000 mean fluorescence intensity, and for the analysis presented we only considered persistent DSA, defined as the same specificity observed in two or more post-transplant sera. Allele-level HLA antibodies such as HLA DQA1*/DQB1* were identified by re-analyzing single antigen bead results compared to the allele-level donor genotype.

### Determining antibody-mediated rejection

All subjects were followed at frequent intervals for assessment of graft function using clinical findings, diagnostic test results (e.g. electrocardiogram and echocardiogram), as well as by serial endomyocardial biopsy performed at the same timepoints indicated for DSA surveillance above and at the time of clinically suspected rejection episodes. For the present study episodes of ABMR were only included when proven by endomyocardial biopsy using local site interpretation, as previously described (19, 21, 22). All patients in the ABMR group that were used for determining HLA molecular mismatch cut-offs had at least one biopsy-proven episode of ABMR.

### Statistical methods

All graphing and statistical analysis were conducted in R version 4.1.2 (https://www.r-project.org/). For correlation statistics the Pearson’s correlation was calculated and the rho (r) value is reported. Quantile analysis was implemented *via* the R stats package. Kaplan-Meier analysis and log-rank tests were performed using the survival and survminer packages. For log-rank tests a p-value of ≤0.05 is considered statistically significant. For Kaplan-Meier analysis all patients were censored at the 1000 days mark because no data were available for any patient past this timepoint. Freedom from DSA was calculated as the time to the first appearance of a persistent, post-transplant DSA. Freedom from ABMR was calculated as the time to the first ABMR diagnosis for a given patient. Data manipulation was accomplished *via* the reshape2 package and base R functions. All confusion matrix statistics were calculated in R using the confusion Matrix function in the caret package.

## Results

### Investigating HLA compatibility using HLA-EMMA

Two different molecular characterizations of donor/recipient HLA compatibility were performed with HLA-EMMA: all mismatches at the amino acid level (AAMM), and solvent-accessible amino acid mismatches (SAAAMM), which are considered to be more “accessible” to anti-HLA antibodies (5). The total AAMM load ranged from 26-193 and SAAAMM load ranged from 18-140 (**Table 2**). We observed a large degree of heterogeneity in this population with respect to AAMM and SAAAMM values, which can also be observed within discrete allele mismatch categories for Total HLA (Class I + Class II; **Figures 1A, D**), Class I (HLA-A+HLA-B+HLA-C; 1B and 1E) and Class II (HLA-DRB1+HLA-DRB3+HLA-DRB4+HLA-DRB5+HLA-DQA1+HLA-DQB1+HLA-DPA1+HLA-DPB1; 1C and 1F).

**Table 2:** Descriptive statistics (N=274).

HLA-EMMA Amino Acid Mismatch	Total	Class I	Class II	DRB1/3/4/5	DQA1/DQB1	DPA1/DPB1
Median	106	44	63	22	28	8
Range (min, max)	167 (26, 193)	82 (0, 82)	153 (0, 153)	60 (0, 60)	78 (0, 78)	25 (0, 25)
IQR	43 (84, 127)	17 (35, 52)	37 (43, 80)	17 (12, 29)	33 (15, 48)	13 (3, 16)
Skewness	0.17	0.14	0.16	0.35	0.22	0.43
HLA-EMMA Solvent-Accessible Amino Acid Mismatch	Total	Class I	Class II	DRB1/3/4/5	DQA1/DQB1	DPA1/DPB1
Median	74	27	47	16	21	6
Range (min, max)	122 (18, 140)	52 (0, 52)	119 (0, 119)	45 (0, 45)	60 (0, 60)	19 (0, 19)
IQR	31 (58, 89)	12 (21, 33)	29 (32, 61)	13 (9, 22)	29 (11, 40)	11 (1, 12)
Skewness	0.2	0.19	0.23	0.35	0.23	0.43

Median, range, interquartile range (IQR), and skewness are listed for HLA-EMMA amino acid mismatch (AAMM) and solvent-accessible amino acid mismatch (SAAAMM). Skewness measures the symmetry of the two tails of a distribution.

### Determining SAAAMM risk cut-offs for DSA and ABMR

**Figure 2:**
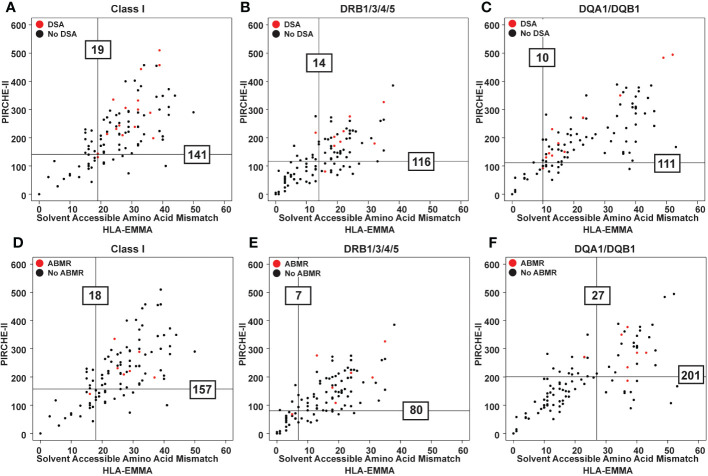
Scatter plots showing PIRCHE-II score vs HLA-EMMA solvent-accessible amino acid mismatch (SAAAMM) score. Data from 100 transplant patients assessed for newly formed post-transplant DSA for each category (Class I, DR, DQ) **(A-C)** and ABMR **(D-F)** development. Datapoints for the patients that experienced the indicated post-transplant clinical event are colored red. Vertical lines represent the SAAAMM cut-off and horizontal lines represent the PIRCHE-II cut-off. Lines were drawn based on quantile analysis (see methods) using the red datapoints. Numbers on the graph indicate the numeric value of the cut-off for developing DSA **(A-C)** or ABMR **(D-F)**.

The 100 transplant cases with clinical data and no pre-formed DSA were used in this study to determine HLA-EMMA SAAAMM cut-offs. Thirty-eight patients (38%) tested positive for DSA post-transplant (29% Class I, 22% Class II, 13% Class I/II), with 26% of DSAs being transient, 26% being persistent, and 14% categorized as both. We only used persistent post-transplant DSA in the following analyses. Among the 26% persistent post-transplant DSA cases the distribution was 9% Class I, 10% Class II, 7% Class I/II, and 7% were diagnosed with biopsy proven ABMR (**Supplementary Tables 1**, **2**). The risk cut-off for developing DSA for each class and specificity was ≥19 for HLA Class I (n=16 patients used to establish cut-off, **Figure 2A**), ≥14 for DRB1/3/4/5 (n=9, **Figure 2B**), and ≥10 SAAAMM for DQA1/DQB1 (n=11, **Figure 2C**). However, the ABMR (n=7) risk cut-off for Class I, DRB1/3/4/5, and DQA1/DQB1 were ≥18 (**Figure 2D**), ≥7 (**Figure 2E**), and ≥27 SAAAMM (**Figure 2F**), respectively. PIRCHE-II risk cut-offs were previously reported on the same dataset and are represented by the horizontal lines shown in **Figure 2** (17).

When examining the distribution of PIRCHE-II scores based on the HLA-EMMA cut-offs, we observed that cases below and above the SAAAMM cut-offs have a wide range of PIRCHE-II scores. Similarly, cases with high or low PIRCHE-II scores have a wide range of SAAAMM values (**Supplementary Table 3**).

### HLA-EMMA helps identifying cases at high risk for DSA and ABMR

**Figure 3:**
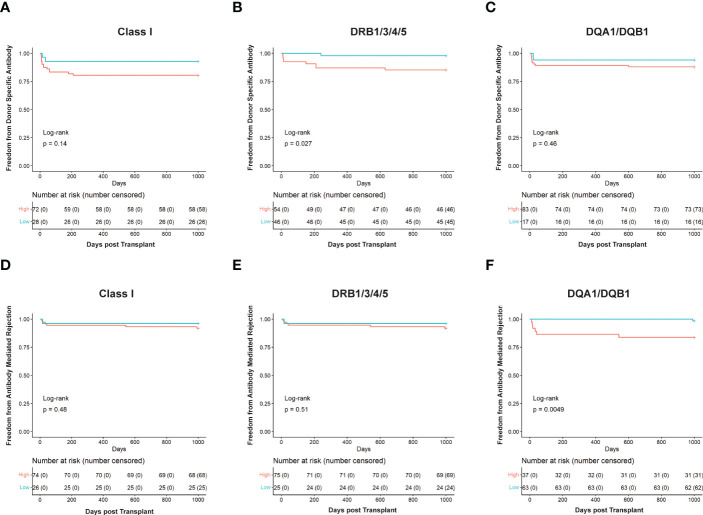
Kaplan Meier survival curves showing risk stratification using cut-offs determined for solvent—accessible amino acid mismatch (SAAAMM) load generated *via* HLA-EMMA. Survival curves for Freedom from donor-specific antibody in Class I **(A)**, DRB1/3/4/5 **(B)**, and DQA1/DQB1 **(C)**, and Freedom from antibody-mediated rejection in Class I **(D)**, DRB1/3/4/5 **(E)**, and DQA1/DQB1 **(F)** were plotted for 100 patients. The blue lines/labels represent low-risk groups, and the red lines/labels represent high-risk groups. Numbers in the table below represent the number of patients in each category at the indicated timepoint.

To further assess the ability of HLA-EMMA to risk stratify cases, we conducted Kaplan Meier analysis to determine freedom from DSA and ABMR (**Figure 3**). Cases with SAAAMM scores above or below the cut-off value were designated as high- or low-risk, respectively. Patients with high-risk for DSA to DRB1/3/4/5 demonstrated significantly lower freedom from DSA (p=0.027, **Figure 3B**). Although not statistically significant, we observed a trend toward lower freedom from DSA in the high-risk groups for Class I (**Figure 3A**) and DQA1/DQB1 (**Figure 3C**). With respect to ABMR, we also observed significantly lower freedom of ABMR in the high-risk group based on DQA1/DQB1 SAAAMM score (p=0.0049; **Figure 3F**). However, the differences in freedom of ABMR between the high- and low-risk groups were diminished when categorized based on Class I (**Figure 3D**) and DRB1/3/4/5 (**Figure 3E**).

### Combining HLA-EMMA SAAAMM and PIRCHE-II scores offers a means to further stratify transplant cases

**Figure 4:**
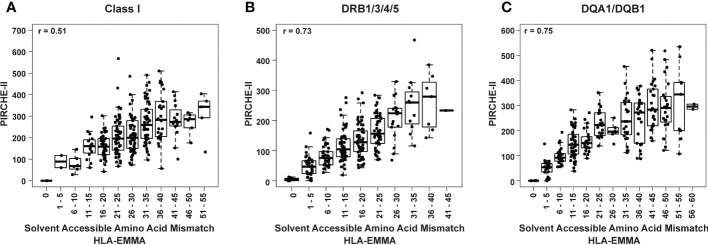
Box and whisker plots of PIRCHE-II score values grouped by HLA-EMMA solvent-accessible amino acid mismatch (SAAAMM) score bins for Class I **(A)**, DRB1/3/4/5 **(B)**, and DQA1/DQB1 **(C)**. Pearson’s correlation coefficient is reported based on correlation of the individual values for PIRCHE-II and SAAAMM.

The two algorithms show correlation values of 0.73 for HLA-DRB1/3/4/5, 0.75 for DQA1/DQB1 and 0.51 for HLA Class I (**Figure 4**). To further examine this, the cohort was divided into discrete incremental categories based on SAAAMM values and PIRCHE-II scores plotted within these categories (**Figure 4**). For each SAAAMM group, large variations in PIRCHE-II score were observed for HLA Class I (**Figure 4A**), DRB1/3/4/5 (**Figure 4B**) and DQA1/DQB1 (**Figure 4C**). For example, cases with a Class I SAAAMM of 21-25 had PIRCHE-II scores ranging from 67 to 568 (**Figure 4A**). For both DSA and ABMR risk, cases with SAAAMM scores above our risk cut-offs for SAAAMM load (**Figure 2**) had PIRCHE-II scores ranging above and below the PIRCHE-II risk cut-off value (**Supplementary Table 3**). Thus, for transplant recipients with high-risk SAAAMM scores, the risk for DSA and ABMR may be further divided based on the PIRCHE-II score cut-off.

### The impact of combining HLA-EMMA and PIRCHE-II scores for risk stratification

To determine if SAAAMM and PIRCHE-II high-risk cases could be re-stratified using a second method, for each predictive algorithm, cases with above cut-off loads were split into intermediate- or high-risk based on the score of the second method (**Table 3**; **Supplementary Figure 1**). As expected, the low-risk cases by individual algorithm or the combined method had the lowest incidence of DSA and ABMR (**Table 3**) and the high-risk cases had the highest incidence (**Table 3**). Interestingly, the intermediate-risk cases, defined as high-risk by one algorithm but low-risk by the other, demonstrated a DSA and ABMR incidence like the low-risk cases (**Table 3**). Although the results of these analyses were not statistically significant, our data appear to indicate that high-risk cases could be further divided into higher and lower risk groups based on the second score. DSA and ABMR risk scores generated from molecular mismatch data for DRB1/3/4/5 (**Figure 3B**) and DQA1/DQB1 (**Figure 3F**), respectively, suggest that patients considered to be high-risk by one algorithm, but low-risk by the other, show a lower tendency to develop DSA and ABMR and could be reclassified as intermediate-risk.

**Table 3:** Frequency of donor-specific antibody (DSA) and antibody-mediated rejection (ABMR) in various high- and low-risk groups defined based on solvent-accessible amino acid mismatch (SAAAMM) load and PIRCHE-II score cut-offs determined in **Figure 2**.

	DSA	Class I	Class I (% DSA)	HLA-DR	HLA-DR (% DSA)	HLA-DQ	HLA-DQ (% DSA)
LOW RISK	Low SAAAMM	28	2 (7.14)	46	1 (2.17)	17	1 (5.88)
	Low PIRCHE-II	24	1 (4.17)	48	1 (2.08)	23	1 (4.35)
	Low SAAAMM & Low PIRCHE-II	18	1 (5.56)	35	0 (0.00)	13	1 (7.69)
INTERMEDIATE RISK	Low SAAAMM & High PIRCHE-II or High SAAAMM & Low PIRCHE-II	16	1 (6.25)	24	2 (8.33)	14	0 (0.00)
HIGH RISK	High SAAAMM	72	14 (19.44)	54	8 (14.81)	83	10 (12.05)
	High PIRCHE-II	76	15 (19.74)	52	8 (15.83)	77	10 (12.99)
	High SAAAMM & High PIRCHE-II	66	14 (21.21)	41	7 (17.07)	73	10 (13.70)
	ABMR	Class I	Class I (% DSA)	HLA-DR	HLA-DR (% DSA)	HLA-DQ	HLA-DQ (% DSA)
LOW RISK	Low SAAAMM	26	1 (3.85)	25	1 (4.00)	63	1 (1.59)
	Low PIRCHE-II	31	1 (3.23)	30	1 (3.33)	60	1 (1.67)
	Low SAAAMM & Low PIRCHE-II	19	1 (5.26)	20	1 (5.00)	49	0 (0.00)
INTERMEDIATE RISK	Low SAAAMM & High PIRCHE-II or High SAAAMM & Low PIRCHE-II	19	0 (0.00)	15	0 (0.00)	25	2 (8.00%
HIGH RISK	High SAAAMM	74	6 (8.11)	75	6 (8.00)	37	6 (16.22)
	High PIRCHE-II	69	6 (8.70)	70	6 (8.57)	40	6 (15.00)
	High SAAApMM & High PIRCHE-II	62	6 (9.68)	65	6 (9.23)	26	5 (19.23)

To determine the impact of using both molecular mismatch scoring methods together, we performed Kaplan Meier analysis after dividing the 100 cases into three discrete categories: high-risk patients with an above cut-off score for both algorithms (top right quadrant of graphs in **Figure 2**), intermediate-risk patients with an above cut-off score by one but not both algorithms (top left and bottom right quadrants of graphs in **Figure 2**), and low-risk patients with below cut-off scores from both algorithms (bottom left quadrant of graphs in **Figure 2**).

**Figure 5:**
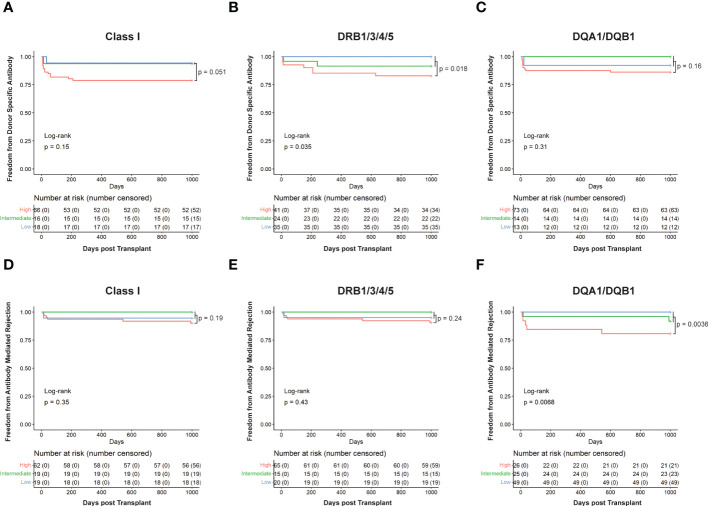
Kaplan Meier survival curves showing patients’ risk stratified using cut-offs determined for HLA-EMMA solvent-accessible amino acid mismatch (SAAAMM) and PIRCHE-II score. Survival curves for Freedom from donor-specific antibody in Class I **(A)**, DRB1/3/4/5 **(B)**, and DQA1/DQB1 **(C)**, and Freedom from antibody-mediated rejection in Class I **(D)**, DRB1/3/4/5 **(E)**, and DQA1/DQB1 **(F)** were plotted for 100 patients. Stratification was performed by placing patients into three risk categories: High) patients with an above cut-off score for both algorithms (red line), Intermediate) patients with an above cut-off score by one of the two algorithms and a below cut-off score for the other (green line), Low) patients with below cut-off scores from both algorithms (blue line). Numbers in the table below each graph represent the number of patients in each category at the indicated timepoint. Log rank p-values are reported for all three risk categories (high, intermediate, and low; bottom left of graphs) or high versus low + intermediate (top right of graph).

When assessing freedom from Class I DSA (**Figure 5A**), low- and intermediate-risk categories behaved similarly while high-risk clearly showed a decreased freedom from DSA. However, DRB1/3/4/5 (**Figure 5B**; p=0.035) and DQA1/DQB1 (**Figure 5C**) showed clear separation between the low, intermediate, and high-risk categories, with the low-risk category having the longest time free of DSA, the high-risk having the least, and the intermediate falling between the two. Using this method, 16 cases for Class I, 24 for DRB1/3/4/5, and 14 for DQA1/DQB1 could be reclassified as intermediate risk. We also performed log-rank test to compare high-risk to a combined low- and intermediate-risk category. This resulted in an increase in statistical significance at DRB1/3/4/5 (p=0.018) and nearly reached statistical significance for Class I (p=0.051), but still failed to reach statistical significance at DQA1/DQB1 (**Figure 5**).

Like the ABMR risk stratification by HLA-EMMA (**Figure 3**) or PIRCHE-II (17) alone, the combined algorithms split the cohort to a lesser extent for Class I (**Figure 5D**) and DRB1/3/4/5 (**Figure 5E**) but did so significantly (p=0.0068) for DQA1/DQB1 molecular mismatch (**Figure 5F**). For ABMR risk, a higher number of cases may be needed to assess the role of combining HLA-EMMA and PIRCHE-II for Class I and DRB1/3/4/5 molecular mismatch; this analysis method will be tested again when the clinical data for the full cohort is available. However, with respect to ABMR risk at DQA1/DQB1, the two-algorithm method allowed us to reclassify 25 cases from high-risk to intermediate-risk. Combining the low- and intermediate-risk groups and comparing them to the high-risk group again led to similar results with a slight decrease in p-value for all HLA loci tested.

To assess the differences between the performance of our method for defining high risk patients and the low or intermediate risk patients we calculated various statistics using a confusion matrix (**Supplementary Table 4**). To do this we combined the low- and intermediate-risk groups into a single category and compared those patients to our high-risk patients. We focused on the negative predictive value (NPV), which tells us the likelihood that a patient below our cutoff does not have DSA or ABMR. We found high NPVs for the combined and individual methodologies when assessing DSA (Class I: SAAAMM = 0.93, PIRCHE-II = 0.96, Combined = 0.94; DRB1/3/4/5: 0.98, 0.97, 0.97; DQA1/DQB1: 0.94, 0.96, 0.96) and ABMR (Class I: 0.96, 0.97, 0.97; DRB1/3/4/5: 0.96, 0.97, 0.97; DQA1/DQB1: 0.98, 0.98, 0.97), meaning that the likelihood that a patient in our low- and intermediate-risk categories does not have DSA or ABMR is extremely high.

## Discussion

In this paper we define three levels of risk (low, intermediate, high) and correlate them with post-transplant DSA and ABMR in pediatric heart transplant recipients. The results demonstrate the usefulness of the two-algorithm method in stratifying risk in a more granular way by defining an intermediate risk cohort. Similar results were published previously using HLAMatchmaker and PIRCHE-II (17).

The value of evaluating HLA mismatch in heart transplantation has been demonstrated in numerous studies (1, 23–25) and HLAMatchmaker, HLA-EMMA, and PIRCHE-II have been described as useful tools for predicting risk and managing patient and graft survival (18). Our study supports these findings and is the first ever to use HLA-EMMA to define risk cut-offs in pediatric heart transplant (**Figure 2**). Our results suggest that HLA-EMMA alone has the capability to risk stratify patients into high- and low-risk groups for DSA and ABMR (**Figure 3**), as does PIRCHE-II (17). We are also the first to use HLA-EMMA and PIRCHE-II together to risk-stratify pediatric heart transplant patients and we demonstrate the true potential of these algorithms by showing that DSA and ABMR outcomes can be better predicted using the combined metrics in this unique population.

The combined method allows us to define high-, intermediate-, and low-risk groups. High-risk patients have above cut-off scores for both PIRCHE-II and HLA-EMMA while low-risk patients have below cut-off scores for both algorithms. Intermediate-risk is the most interesting category for illustrating the logic behind the combined scoring method, having been assigned an above cut-off score by one algorithm and a below cut-off score by the other. This situation represents two possible outcomes: 1) if PIRCHE is above cut-off and HLA-EMMA is below, T-cell activation is likely to occur, but the B-cell response may be lacking, or 2) if HLA-EMMA is above cut-off and PIRCHE-II is below, T-cell activation, a prerequisite for B-cell response, may not occur and because of this the B-cell response will be limited. Therefore, we believe that this newly defined intermediate category contains lower risk patients similar to the low-risk group that shows a low incidence of DSA and ABMR.

Our findings suggest that HLA-DRB1/3/4/5 mismatches are more important for DSA development than HLA-DQA1/DQB1 (**Figures 3**, **5**). This fits with a recent report demonstrating that HLA-EMMA derived AAMM loads at HLA-DRB1/3/4/5 were shown to be associated with worsening graft survival in heart transplant patients (26) High HLA-DR/DQ mismatch was reported to put heart transplant patients at higher risk of late graft loss (25). The trend at HLA-DQA1/DQB1 mismatches appears less significant in our data (**Figure 3C**, **5C**); however, the high-risk group still experiences worse outcomes (**Figure 5C**), suggesting that we should not discount it. Indeed, previous studies have identified HLA-DQ as an important factor for the development of DSA in heart transplant patients (23, 25). Class I molecular mismatch scoring also appears to be important (**Figures 4A**, **5A**), but failed to reach statistical significance in this study, although low- and intermediate-risk groups are experiencing far better DSA outcomes than the high-risk group (**Figure 5A**). Poor Class I (HLA-A/B) HLA matching has also been shown to lead to worse long term graft survival (25). Similar outcomes were observed for Class I, HLA-DR, and HLA-DQ, in our previous study combining the HLAMatchmaker and PIRCHE-II algorithms, although most failed to reach statistical significance (17).

We found that ABMR risk predictions were best when using data from HLA-DQA1/DQB1 molecular matching (**Figures 3F**, **5F**). In fact, when using data from HLA-DRB1/3/4/5 or Class I (**Figures 3D, E**, **5D, E**) we observe little difference between high- and low-risk populations in the HLA-EMMA analysis and the combined method. However, more data is required to obtain the statistical power necessary to fully assess Class I and DRB1/3/4/5 molecular mismatch in this context. The fact that HLA-EMMA and PIRCHE-II scores suggest that HLA-DQA1/DQB1 mismatch is the strongest indicator of ABMR is in line with previous findings in kidney transplantation. In kidneys, HLA-DQ mismatch and anti-DQ DSA are known to be one of the most important factors in the development of ABMR (27).

Interestingly, our cohort’s HLA-EMMA HLA-DQ results (x-axis values in **Figure 2C, F**) show that the SAAAMM data is bimodally distributed. We do not observe this type of distribution at HLA-DQ by PIRCHE-II or HLAMatchmaker (17), nor do we observe it by any method at Class I or HLA-DR.

Our future work will continue to pursue the use of multiple methods of HLA molecular mismatch to better predict risk. Determining the immunogenicity of the amino acid substitutions and the eplets they compose, and how best to utilize this additional information will be essential. Learning how to employ these methods in the clinical setting will be a challenge, but we believe it has great potential to advance personalized approaches of patient management. Assessment of possibilities such as a composite score or simply reporting an ensemble of scores, as we have here, will be necessary to determine which will be the most useful in practice.

## Limitations

This work is limited by the size of the cohort used for clinical correlations (n=100), which results in our statistics being underpowered throughout. We also note that as we split these data into groups to categorize them into high, intermediate, and low risk we occasionally end up with small subsets, for example 13/100 at low risk for DSA at HLA-DQ. The fact that we can achieve statistical significance given the constraints of our dataset speaks to the predictive value of the HLA molecular mismatch algorithms tested. It is important to note that in pediatric heart transplant this cohort is the largest ever assembled with high quality HLA typing results. Importantly, we have obtained the most accurate HLA genotyping information available and do not impute any genotypic information. Since the size of the cohort is limiting in terms of statistical power, we present these results as a pilot study to be followed up by additional analysis when clinical outcome data become available for our additional 143 patients.

Another limitation may involve the role of non-HLA antibodies in pediatric heart transplantation and how it may impact the accuracy of risk assessment for ABMR using the HLA molecular mismatch platforms. Although the major role of pre-formed and *de novo* HLA antibodies on the development of ABMR has been well established, approximately one-third of histologically diagnosed ABMR lack circulating donor specific HLA antibody (DSA) (28–30). Other targets on endothelial cells, some polymorphic such as major histocompatibility complex class I chain-related antigens A (MICA), or auto-antibodies such as vimentin, myosin and angiotensin II type1 receptor AT1R (31–34) have been documented in ABMR cases that lack circulating DSA. However, the most detrimental effects on allografts were observed in the presence of both HLA and non-HLA antibodies (35). The association of HLA mismatches and histology suggestive of ABMR in absence of DSA has been studied in 121 renal transplant recipients using multiple tools such as HLAMatchmaker, PIRCHE-II and HLA- EMMA (29). The study indicated that the lesions associated with histology of ABMR are not specific to antibody involvement and can be caused by immune activation through donor-recipient HLA mismatch (29). In contrast, Crespo et al. (2021) (36) showed that total Class II and DRB1 HLA epitope mismatch were associated with DSA-positive ABMR but not with DSA-negative ABMR in a limited number (n=3) of renal transplant recipients (36). Interestingly, we also observed in our study that 2/7 ABMR cases were diagnosed in the absence of circulating DSA, however, we found similar distribution of HLA molecular mismatch scores as shown for the ABMR cases with DSA (**Supplementary Table 2**). Since ABMR diagnosis was within 6 weeks post-transplant it may reflect memory responses of either low level of DSA absorbed by the graft or of pre-formed non-HLA antibody. These findings will be further validated when we have access to the clinical findings of the entire pediatric cohort from CTOT.

In addition, the current analysis produces 6 scores for each donor-recipient comparison. We would like to simplify this into a single risk assessment to make it more useful however more work is required before this can be implemented. This study focused on DSA and ABMR, but we are aware that additional immunological variables should be considered before applying this score system for risk stratification and individualized patient management.

## Conclusion

We have demonstrated that HLA-EMMA and PIRCHE-II can be used to risk stratify patients based on DSA and ABMR outcomes. More importantly, when both methods are used together, we were able to further stratify our population and identify low-, intermediate-, and high-risk groups for the development of DSA and ABMR. We believe that this type of risk stratification, validated by larger cohorts, will improve individualized patient management.

## Data availability statement

The datasets presented in this article are not readily available because it is part of a larger dataset that was not released/completed by NIH supported clinical trial NCT02752789. Requests to access the datasets should be directed to ellisoniima@upmc.edu.

## Author contributions

All authors listed have made a substantial, direct, and intellectual contribution to the work and approved it for publication.
