# Supplementary material for: Immunologic risk stratification of pediatric heart transplant patients by combining HLA-EMMA and PIRCHE-II
Source: Front Immunol. 2023 Mar 14;14:1110292. doi: 10.3389/fimmu.2023.1110292 (PMC10043167; doi:10.3389/fimmu.2023.1110292)
Supplement: Supplementary file 1 [file DataSheet_1.pdf]

**SUPPLEMENTARY MATERIAL**

Supplementary Table 1. All cases with persistent post-transplant DSA showing DSA specificity and molecular mismatch scores from HLA-EMMA and PIRCHE-II at Class I, DR, and DQ. Days to first DSA identification are listed. In some cases DSAs are identified on different days and are labeled with (I) for Class I, (DR) HLA-DRB1/3/4/5, or (DQ) HLA-DQA1/DQB1.

| | | | | **SAAAMM** | | | **PIRCHE-II** | | |
|---|---|---|---|---|---|---|---|---|---|
| **Tx Pair ID** | **Days to DSA** | **DSA Specificity** | **DSA Summary** | **Class I** | **DR** | **DQ** | **Class I** | **DR** | **DQ** |
| Pair 16 | 34 | A*02:01, DQB1*02:01/ DQA1*05:01 | Class I (1), DQA1/DQB1 (1) | 19 | 18 | 17 | 131 | 84 | 150 |
| Pair 19 | 14 | DRB5*01:02 | DRB5 (1) | 32 | 16 | 36 | 173 | 80 | 143 |
| Pair 21 | 8 | B*08:01 | Class I (1) | 28 | 21 | 27 | 306 | 147 | 212 |
| Pair 34 | 9 | B*45*01 | Class I (1) | 26 | 12 | 1 | 243 | 68 | 8 |
| Pair 35 | 150 (DR), 14 (DQ) | DRB4*01:03, DQB1*03:02/ DQA1*03:03 | DRB4 (1), DQA1/DQB1 (1) | 34 | 19 | 11 | 457 | 171 | 130 |
| Pair 37 | 180 | C*04:01 | Class I (1) | 36 | 18 | 11 | 288 | 272 | 193 |
| Pair 44 | 12 | A*24:02, B*45:01 | Class I (2) | 25 | 5 | 37 | 231 | 68 | 187 |
| Pair 45 | 60 (I), 600 (DQ) | B*44:02, DQB1*03:01/ DQA1*03:01, DQB1*03:02/ DQA1*03:03 | Class I (1), DQA1/DQB1 (2) | 32 | 4 | 13 | 332 | 75 | 230 |
| Pair 46 | 14 (I) 25 (DQ) | B*07:02, DQB1*03:01/ DQA1*04:01 | Class I (1), DQA1/DQB1 (1) | 19 | 6 | 15 | 143 | 52 | 180 |
| Pair 48 | 7 | B*18:01, A*31:01 | Class I (2) | 37 | 31 | 43 | 198 | 198 | 286 |
| Pair 50 | 14 | DQB1*03:02/ DQA1*03:01 | DQA1/DQB1 (1) | 6 | 24 | 12 | 28 | 198 | 145 |
| Pair 52 | 630 | DRB1*04:01 | DRB1 (1) | 16 | 21 | 34 | 100 | 186 | 277 |
| Pair 62 | 9 | A*68:02, C*03:04 | Class I (2) | 32 | 3 | 9 | 300 | 47 | 87 |
| Pair 63 | 12 | A*33:03, B*40:06, B*15:12, C*03:03, C*03:02, DQB1*05:02/ DQA1*01:02 | Class I (5), DQA1/DQB1 (1) | 24 | 13 | 35 | 335 | 276 | 350 |

| | | | | | | | | | |
|---|---|---|---|---|---|---|---|---|---|
| Pair 66 | 12 | DRB4*01:03, DQB1*05:02/ DQA1*01:02, DQB1*03:03/ DQA1*03:02 | DRB4 (1), DQA1/DQB1 (2) | 29 | 35 | 23 | 221 | 326 | 271 |
| Pair 67 | 22 (I), 10 (DR) | A*68:03, DRB4*01:03 | Class I (1), DRB4 (1) | 22 | 19 | 10 | 213 | 203 | 120 |
| Pair 77 | 45 | A*03:01, A*01:01, B*08:01, B*50:01 | Class I (4) | 27 | 19 | 40 | 209 | 108 | 286 |
| Pair 80 | 12 | DQB1*02:02/ DQA1*03:01 | DQA1/DQB1 (1) | 32 | 38 | 49 | 368 | 385 | 483 |
| Pair 82 | 22 (I), 240 (DR) | A*02:01, DRB1*15:01 | Class I (1), DRB1 (1) | 39 | 13 | 36 | 457 | 218 | 361 |
| Pair 86 | 9 | A*03:01 | Class I (1) | 31 | 18 | 19 | 238 | 170 | 184 |
| Pair 99 | 210 | A*02:01, B*44:10, B*58:02, DRB4*01:01 | Class I (3), DRB4 (1) | 39 | 24 | 23 | 510 | 275 | 270 |
| Pair 102 | 60 | A*68:01 | Class I (1) | 33 | 6 | 7 | 443 | 171 | 112 |
| Pair 115 | 9 | DQB1*03:01/ DQA1*05:05 | DQA1/DQB1 (1) | 28 | 18 | 13 | 218 | 78 | 137 |
| Pair 116 | 201 (DR), 9 (DQ) | DRB4*01:03, DQB1*03:02/ DQA1*03:01 | DRB4 (1), DQA1/DQB1 (1) | 22 | 22 | 52 | 272 | 223 | 494 |
| Pair 117 | 9 | DRB4*01:03 | DRB4 (1) | 10 | 32 | 16 | 71 | 179 | 105 |
| Pair 126 | 21 | DQB1*03:01/ DQA1*03:03 | DQA1/DQB1 (1) | 27 | 11 | 10 | 135 | 39 | 92 |

Supplementary Table 2. All cases with ABMR confirmed by endomyocardial biopsy. Days to first ABMR diagnosis are listed. When persistent post-transplant DSA was present, we state DSA specificity. Molecular mismatch scores from HLA-EMMA and PIRCHE-II at Class I, DR, and DQ are shown for all.

| | | | | SAAAMM | | | PIRCHE-II | | |
|---|---|---|---|---|---|---|---|---|---|
| **Tx Pair ID** | **Days to ABMR** | **DSA Specificity** | **DSA Summary** | **Class I** | **DR** | **DQ** | **Class I** | **DR** | **DQ** |
| Pair 44 | 17 | A*24:02, B*45:01 | Class I (2) | 25 | 5 | 37 | 231 | 68 | 187 |
| Pair 48 | 26 | B*18:01, A*31:01 | Class I (2) | 37 | 37 | 48 | 198 | 198 | 286 |
| Pair 63 | 11 | A*33:03, B*40:06, B*15:12, C*03:03, C*03:02, DRB1*16:02, DQB1*05:02/ DQA1*01:02 | Class I (5), DRB1 (1), DQA1/DQB1 (1) | 24 | 14 | 35 | 335 | 276 | 350 |
| Pair 66 | 991 | A*02:01, DRB4*01:03, DQB1*05:02/ DQA1*01:02, DQB1*03:03/ DQA1*03:02 | Class I (1), DRB4 (1), DQA1/DQB1 (2) | 29 | 53 | 23 | 221 | 326 | 271 |
| Pair 77 | 542 | A*03:01, A*01:01, B*08:01, B*50:01 | Class I (4) | 27 | 27 | 40 | 209 | 108 | 286 |
| Pair 100 | 41 | none reported | none reported | 32 | 45 | 37 | 289 | 215 | 377 |
| Pair 137 | 15 | none reported | none reported | 16 | 20 | 70 | 140 | 162 | 234 |

Supplementary Table 3. SAAAMM load and PIRCHE-II score ranges for high and low SAAAMM load and PIRCHE-II score groups based on cut-offs defined in Figure 2.

| Molecular Mismatch Score: Outcome | below SAAAMM cut-off (N) | below SAAAMM cut-off PIRCHE-II (range) | above SAAAMM cut-off (N) | above SAAAMM cut-off PIRCHE-II (range) | high SAAAMM and low PIRCHE-II (N) |
|---|---|---|---|---|---|
| Class1: DSA | 28 | 0 to 229 | 72 | 74 to 510 | 6 |
| HLA-DR: DSA | 46 | 0 to 276 | 54 | 68 to 385 | 13 |
| HLA-DQ: DSA | 17 | 0 to 193 | 83 | 51 to 494 | 10 |

| Molecular Mismatch Score: Outcome | below PIRCHE-II cut-off (N) | below PIRCHE-II cut-off SAAAMM (range) | above PIRCHE-II cut-off (N) | above PIRCHE-II cut-off SAAAMM (range) | high PIRCHE-II and low SAAAMM (N) |
|---|---|---|---|---|---|
| Class1: DSA | 24 | 0 to 41 | 76 | 15 to 50 | 10 |
| HLA-DR: DSA | 48 | 0 to 33 | 52 | 6 to 38 | 11 |
| HLA-DQ: DSA | 23 | 0 to 51 | 77 | 7 to 53 | 4 |

| Molecular Mismatch Score: Outcome | below SAAAMM cut-off (N) | below SAAAMM cut-off PIRCHE-II (range) | above SAAAMM cut-off (N) | above SAAAMM cut-off PIRCHE-II (range) | high SAAAMM and low PIRCHE-II (N) |
|---|---|---|---|---|---|
| Class1: ABMR | 26 | 0 to 229 | 74 | 74 to 510 | 12 |
| HLA-DR: ABMR | 25 | 0 to 171 | 75 | 39 to 385 | 10 |
| HLA-DQ: ABMR | 63 | 0 to 350 | 37 | 89 to 494 | 11 |

| Molecular Mismatch Score: Outcome | below PIRCHE-II cut-off (N) | below PIRCHE-II cut-off SAAAMM (range) | above PIRCHE-II cut-off (N) | above PIRCHE-II cut-off SAAAMM (range) | high PIRCHE-II and low SAAAMM (N) |
|---|---|---|---|---|---|
| Class1: ABMR | 31 | 0 to 41 | 69 | 15 to 50 | 7 |
| HLA-DR: ABMR | 30 | 0 to 25 | 70 | 1 to 38 | 5 |
| HLA-DQ: ABMR | 60 | 0 to 53 | 40 | 11 to 52 | 14 |

Supplementary Table 4. Confusion matrix statistics for SAAAMM, PIRCHE-II and combined cutoffs. Statistics presented were calculated based on a confusion matrix generated using the cutoffs presented in Figure 3 for HLA antigens and conditions using the following nomenclature to categorize samples TP (true positive), TN (true negative), FP (false positive), and FN (false negative). Statistics are calculated as follows: Accuracy ([TP+TN]/[TP+TN+FP+FN]), Sensitivity (TP/[TP+FN]), Specificity (TN/[TN+FP]), Positive Predictive Value (TP/[TP+FP]), Negative Predictive Value (TN/[TN+FN]), Prevalence ([TP+FN]/[ TP+TN+FP+FN]), and Detection Rate (TP/[TP+TN+FP+FN]).

| | **DSA** | | | | | | | | |
|---|---|---|---|---|---|---|---|---|---|
| | **Class I** | | | **DR** | | | **DQ** | | |
| **Statistic** | **SAAAMM** | **PIRCHE-II** | **Both** | **SAAAMM** | **PIRCHE-II** | **Both** | **SAAAMM** | **PIRCHE-II** | **Both** |
| Accuracy | 0.40 | 0.38 | 0.46 | 0.53 | 0.55 | 0.64 | 0.26 | 0.32 | 0.36 |
| Sensitivity | 0.88 | 0.94 | 0.88 | 0.89 | 0.89 | 0.78 | 0.91 | 0.91 | 0.91 |
| Specificity | 0.31 | 0.27 | 0.38 | 0.49 | 0.52 | 0.63 | 0.18 | 0.25 | 0.29 |
| Positive Predictive Value | 0.19 | 0.20 | 0.21 | 0.15 | 0.15 | 0.17 | 0.12 | 0.13 | 0.14 |
| **Negative Predictive Value** | **0.93** | **0.96** | **0.94** | **0.98** | **0.98** | **0.97** | **0.94** | **0.96** | **0.96** |
| Prevalence | 0.16 | 0.16 | 0.16 | 0.09 | 0.09 | 0.09 | 0.11 | 0.11 | 0.11 |
| Detection Rate | 0.14 | 0.15 | 0.14 | 0.08 | 0.08 | 0.07 | 0.10 | 0.10 | 0.10 |

| | **ABMR** | | | | | | | | |
|---|---|---|---|---|---|---|---|---|---|
| | **Class I** | | | **DR** | | | **DQ** | | |
| **Statistic** | **SAAAMM** | **PIRCHE-II** | **Both** | **SAAAMM** | **PIRCHE-II** | **Both** | **SAAAMM** | **PIRCHE-II** | **Both** |
| Accuracy | 0.31 | 0.36 | 0.43 | 0.30 | 0.35 | 0.40 | 0.68 | 0.65 | 0.77 |
| Sensitivity | 0.86 | 0.86 | 0.86 | 0.86 | 0.86 | 0.86 | 0.86 | 0.86 | 0.71 |
| Specificity | 0.27 | 0.32 | 0.40 | 0.26 | 0.31 | 0.37 | 0.67 | 0.63 | 0.77 |
| Positive Predictive Value | 0.08 | 0.09 | 0.10 | 0.08 | 0.09 | 0.09 | 0.16 | 0.15 | 0.19 |
| **Negative Predictive Value** | **0.96** | **0.97** | **0.97** | **0.96** | **0.97** | **0.97** | **0.98** | **0.98** | **0.97** |
| Prevalence | 0.07 | 0.07 | 0.07 | 0.07 | 0.07 | 0.07 | 0.07 | 0.07 | 0.07 |
| Detection Rate | 0.06 | 0.06 | 0.06 | 0.06 | 0.06 | 0.06 | 0.06 | 0.06 | 0.05 |

## Supplementary Figure 1

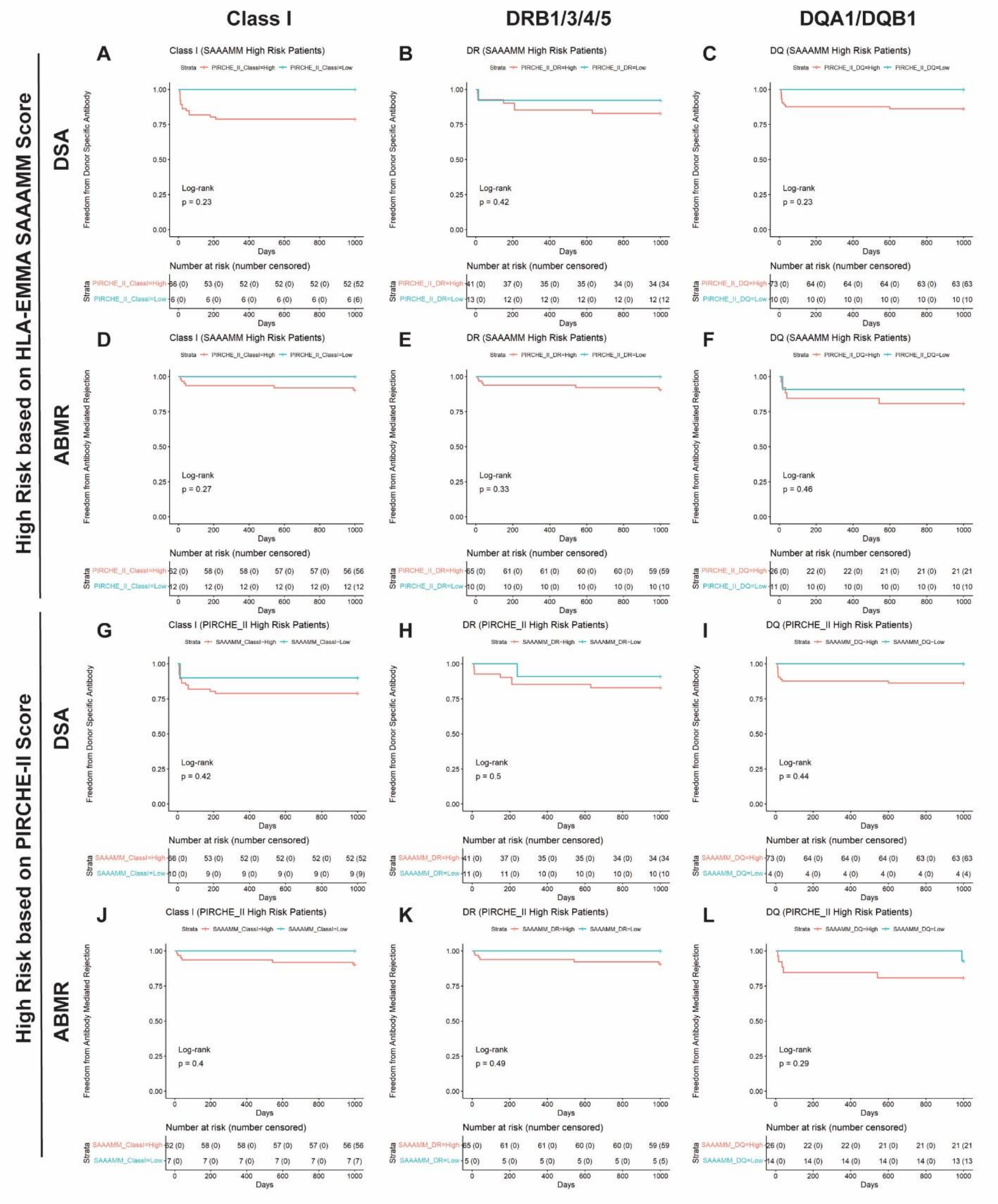


Supplementary Figure 1. Kaplan Meier survival curves showing further risk stratification achieved by HLA-EMMA SAAAMM and PIRCHE-II. Patients considered high risk by SAAAMM load (A-F) or PIRCHE-II score (G-L) are considered in this analysis. These high-risk patients were then re-stratified by either PIRCHE-II score (A-F) or SAAAMM load (G-L), respectively, and Kaplan-Meier analysis was conducted. (A-

C and G-I) Freedom from donor-specific antibody (DSA) in Class I (A), DRB1/3/4/5 (B), and DQA1/DQB1 (C) for re-stratified high-risk patients. (D-F and J-L) Freedom from antibody-mediated rejection (ABMR) in Class I (D), DRB1/3/4/5 (E), and DQA1/DQB1 (F) for re-stratified high-risk patients. Cut-offs presented in Figure 2 were used for this analysis.
